# Pen Wipings

**Published:** 1869-04

**Authors:** 


### Pen Wipings.

BY R. A. H.

"*How not to be Sick*"—by Dr. ALBERT BELLOWS, and published by Hurd & Houghton, is a sequel to his "*Philosophy of Eating*."

The book is a valuable one for many people—for those who are always in a state of uncertainty as regards their diet and clothing and treatment of little ailments. The chapters bearing upon these subjects are deserving of high confidence and consideration. The book is good sense put into good language.

That part of the book which treats upon homeopathy is debatable, and must be judged by every one's own mind upon that subject. Get the book and read it.

*The Annual of Scientific Discovery*, for 1869, issued by Gould & Lincoln, is well worth the attention of many who are perhaps ignorant of its publication. In it, are all the important additions of the year to the scientific world. Its pages preserve and bring to them facts of far greater value than the cost of the book, many times over. It is a grand thing for School District Libraries.

—How many farmers have ever tried to conduct their farms in a regular business-like manner, by opening an account with all parts of the farm—charging and crediting labor and fruits?

Let a farmer for instance, give each field a number, and when the spring comes charge to that field every expense put upon it, and in the autumn credit its products at their market value. Thus he will know if he has made a fair investment upon the field. Or, let him charge his animals in a like manner, with food and care, and credit the receipts of its kind as received.—He will soon learn what are his profitable crops and which are his valuable animals. When this is ascertained, let him dispose of the unremunerative stock of every kind. There are many exceedingly useful books, with forms all ready prepared and blanks to be filled, suggesting to any one of thought, the easy methods of making definite and accurate minutes as the seasons advance, until the end of the year calls for net results. Won't some of our good farmer friends get one of these "helps" and try the *systematic* way of farming.

—We wish to say a good word for "*The Galaxy*," which has now been before the world for three years, and been constantly improving in every respect. That it is popular is attested by the fact that the publishers announce that the subscription in the month of December was more than doubled. We call it a *first class literary magazine.*

—A "Great National Peace Jubilee and Musical Festival" is to be held in Boston in June. A huge temporary building is to be put up on the Common, to hold fifty thousand people. The Jubilee is to begin at noon on the 15th of June, by prayer and the delivery of addresses of various characters. The musical programme will be opened with "Hail Columbia," as follows:

*Symphony*—"Hail Columbia" once through, by the full band of 1000 performers.

*First Verse*—Full band of 1000, and grand chorus of 20,000.

*Second Verse*—Full band; grand chorus and chiming of all the bells in the city.

*Third Verse*—Full band of 1000, grand chorus of 20,000, bells chiming, drums rolling, infantry firing and cannon pealing in the distance, in exact time with the music—the bells rung and cannon fired by electricity from the music stand.

The second day is to be a grand classical programme.

On the third day, the 17th, the anniversary of the battle of Bunker Hill, the culmination is reached. Various selections are to be made, among them the "Anvil Chorus."

The grand chorus of 20,000 and full chorus of 1,000, one hundred anvils, several drum corps, artillery, infantry, bells, &c., are to be introduced. One hundred members of the Fire Department are to do the anvil work.

We don't believe the noise, or music, either one, can be heard at this distance. Elmira is too far off, fortunately, to have its inhabitants deafened by too much music.

